# 

**Published:** 1896-09

**Authors:** 


### CONTENTS:

### SELECTIONS.

Article I.—A Plea for the Higher Scientific Education of the Individual Members of the Profession. By Wm. A. Mills, D. D S. - - - - - - - 193

" II.—Some Suggestions on Bridge-work. By Frank L. Platt, D. D. S. - - - - - - - 199

" III.—"Borism," or the Toxic Effects of Borax. - - 204

" IV.—Cataphoresis. By W. W. Moorehead, D. D. S. - 206

" V.—The Physical Characteristics of the Teeth. By Thos. Fletcher, E. C. S. - - - - - 213

" VI.—Gold Fillings. F. H. Skinner, D. D. S - - 215

" VII.—The Successful Dentist. By C. E. Bently, C. D. S. 217

" VIII.—The Future of Dentistry. By Dr. F. F. Fletcher. - 220

" IX.—Dark Joints in Vulcanite Plates—How to Prevent Same. - - - - - - - - 225

" X.—Treatment of Decay in Proximate Surfaces of Bicuspids and Molars. By Julius G. W. Werner, D. M. D. - - - - - - - - 226

### EDITORIAL, ETC.

The Philadelphia Polyclinic. - - - - - - - 232

Prevention of Infectious Diseases. - - - - - - 233

### MONTHLY SUMMARY.

Medical Hints. - - - - - - - - - 234

The Life and Growth of the Tooth. - - - - - - 235

C. D. Hertz, in Items of Interest. - - - - - - 236

A Local Obtundent. - - - - - - - - 236

Method of Verifying the Quality of Alcohol. - - - - 237

How to Disguise the Taste of Cod Liver Oil. - - - - 237

For Separating Teeth. - - - - - - - - 237

Cuspid Angles. - - - - - - - - - 238

A Remedy for Thirst. - - - - - - - - 238

Bacteriologists. - - - - - - - - - 239

With a Poisoned Lancet. - - - - - - - - 239

For Sensitive Dentine. - - - - - - - - 239

Root Sterilization. - - - - - - - - - 240

A Half Century Mark in Dental Education. - - - - 240

A Substitute for Gold. - - - - - - - - 240

